# Multi-color fluorescent reporter dengue viruses with improved stability for analysis of a multi-virus infection

**DOI:** 10.1371/journal.pone.0194399

**Published:** 2018-03-16

**Authors:** Amporn Suphatrakul, Thaneeya Duangchinda, Natapong Jupatanakul, Kanjanawadee Prasittisa, Suppachoke Onnome, Jutharat Pengon, Bunpote Siridechadilok

**Affiliations:** National Center for Genetic Engineering and Biotechnology, Klong Luang, Pathumthani, Thailand; University of Texas Medical Branch at Galveston, UNITED STATES

## Abstract

Reporter virus is a versatile tool to visualize and to analyze virus infections. However, for flaviviruses, it is difficult to maintain the inserted reporter genes on the viral genome, limiting its use in several studies that require homogeneous virus particles and several rounds of virus replication. Here, we showed that flanking inserted GFP genes on both sides with ribosome-skipping 2A sequences improved the stability and the consistency of their fluorescent signals for dengue-virus-serotype 2 (DENV2) reporter viruses. The reporter viruses can infect known susceptible mammalian cell lines and primary CD14+ human monocytes. This design can accommodate several fluorescent protein genes, enabling the generation of multi-color DENV2-16681 reporter viruses with comparable replication capabilities, as demonstrated by their abilities to maintain their fluorescent intensities during co-infections and to exclude superinfections regardless of the fluorescent tags. The reported design of multi-color DENV2 should be useful for high-throughput analyses, single-cell analysis, and characterizations of interference and superinfection in animal models.

## Introduction

Flaviviruses are a constant threat to global public health, with re-emerging outbreaks of yellow fever [1], new threats from Zika [2], and recurrent outbreaks of dengue in various countries [3]. Flaviviruses are positive-sense RNA viruses with non-segmented, single-stranded RNA genome with the size of approximately 10–12 kb [4]. Several flaviviruses are human pathogens transmitted by arthopods such as mosquitoes and ticks [5]. Many tools and innovative techniques have been employed to dissect flavivirus replication, transmission, and evolution.

Reporter virus has been a versatile tool to visualize and analyze virus infection. Signal intensity from the reporter provides a convenient measurement of virus replication for high-throughput assays and screens. With advances in single-cell sequencing, fluorescent reporter virus in combination with fluorescent-activated cell sorting (FACS) can be used to isolate target cells for molecular profiling [6]. Bioluminescent reporter virus can serve as a sensitive probe to track virus infection in animal models [7]. Several studies have reported the construction of reporter flaviviruses [8–13]. These studies have demonstrated the difficulty of maintaining a reporter gene on the flavivirus genome as it was often quickly deleted after only a few passages of virus in cultured cells. The instability of the reporter gene on viral genome could hamper the use of the reporter virus in many studies that require relatively homogeneous virus preparation and that involve multiple rounds of virus replication such as persistent infection and transmission.

Here, we describe a reporter design in which a reporter gene was inserted at the start of viral open reading frame. Ribosome-skipping 2A sequence flank the reporter gene on both sides, which we show are necessary for the stability of the reporter gene on the Dengue virus type 2 (DENV2) genome. The expression of a GFP separated from the viral proteins by ribosome skipping also generated consistent fluorescent distribution signal in infected cells, as shown using different GFP genes. This design could accommodate several fluorescent genes, enabling the generation of a panel of multi-color DENV2 reporter viruses with comparable replication abilities. In addition to mammalian cell lines that supported DENV replication, the fluorescent reporter viruses could infect human CD14+ monocytes through the mechanism of antibody-dependent enhancement (ADE). We demonstrated the potential of multi-color DENV reporter viruses in the analyses of multi-virus infections by co-infections and superinfection exclusions.

## Results

**Fig 1:**
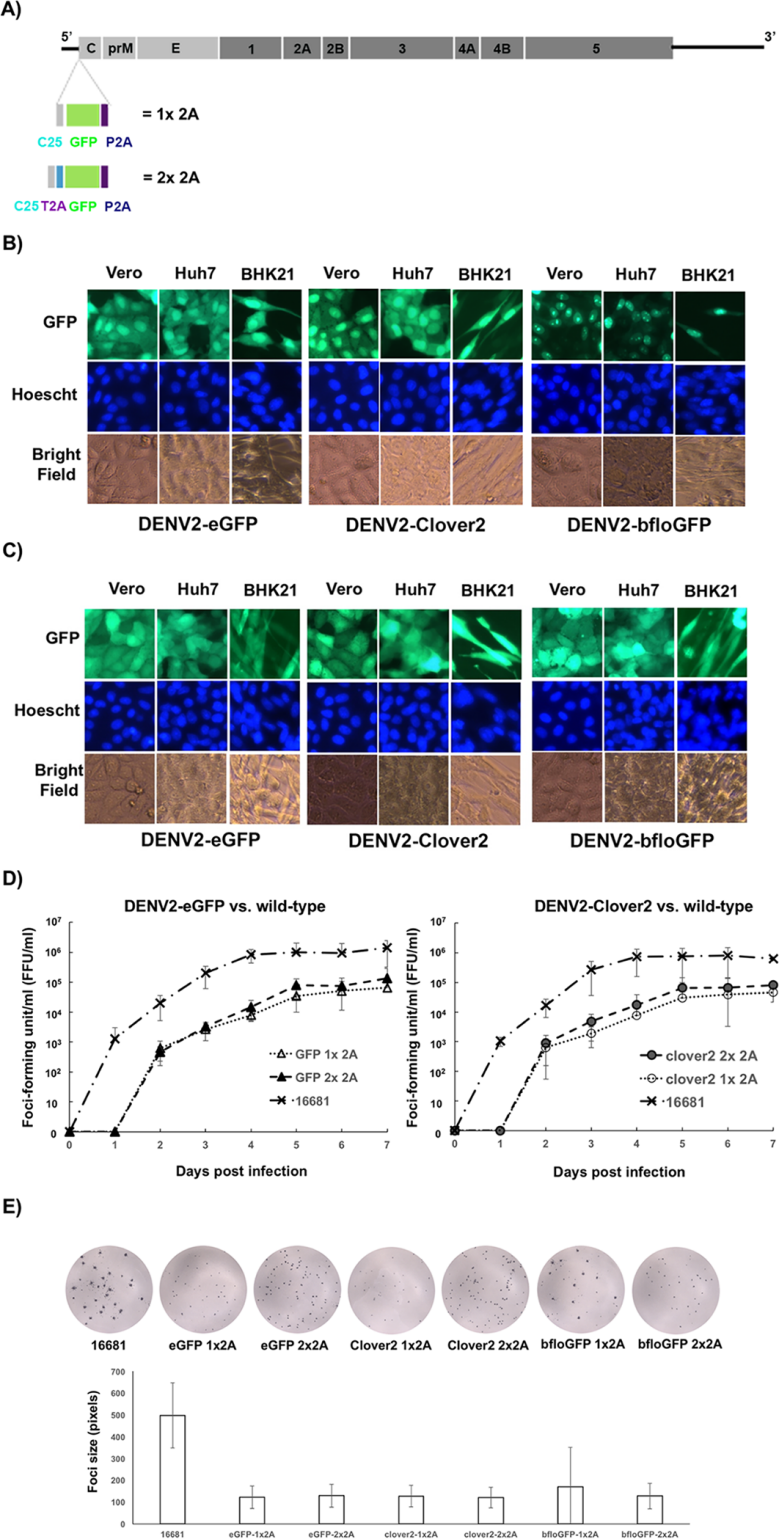
Improved consistency of cellular fluorescent distribution after separation of GFP genes from C25 by 2A ribosome skipping. A) Design of FP reporter gene insertion in the DENV2 genome. P2A = 2A sequence of porcine technovirus-1. T2A = 2A sequence of Thoseasigna virus. 1x 2A denotes the reporter DENV2 design with one 2A sequence at the end of the reporter gene, while 2x 2A denotes the design with two 2A sequences flanking the reporter gene. B) Fluorescent images of Vero, Huh7, and BHK21 cells infected with DENV2-FPs with the 1X 2A design. C) Fluorescent images of Vero, Huh7, and BHK21 cells infected with DENV2-FPs with the 2X 2A design. D) Replication kinetics of DENV2-eGFP (right) and DENV2-clover2 (left) with 1x 2A and 2x 2A designs in Vero cells. Control DENV2 virus without a reporter gene and 2A sequences is denoted as 16681. Confluent Vero cells were infected with one virus at the multiplicity of infection (MOI) = 0.01. Infectious virus titer in the media was quantified by focus-forming assay. Each point is the average of three replicates with error bar representing the standard deviation. E) Focus images of the wild-type and the reporter DENV2-FPs (top panel) and the bar graph comparing the sizes of their foci (bottom panel). The size of each focus was quantified by the number of pixels that it occupied on a digitized image taken on ELISpot reader. Each bar represents the average of the measured focus sizes and error bars represent the standard deviation. At least 227 foci of each virus were used for foci-size quantification.

Our initial effort to generate a reporter GFP virus of DENV2 strain 16681 entailed the expression of enhanced green fluorescent protein (eGFP) fused to the first twenty-five amino acids of capsid (C25) at its N-terminus. The reporter protein cannot be expressed from the 5′ terminus of the viral genome since C25 is needed for translation initiation of dengue virus [14]. We used the same strategy for reporter expression described in [10], in which ribosome-skipping 2A sequence from porcine technovirus-1 (P2A) is expressed C-terminal to the reporter protein (denoted as 1x 2A in Fig 1A). The 2A sequence causes the ribosome to skip formation of a peptide bond during protein synthesis, resulting in the separation between the polypeptides upstream and downstream of the 2A sequence [15]. In this design, reporter protein is expressed as a separate polypeptide from virus proteins and does not interfere with their functions. P2A was chosen instead of the 2A sequence from foot-mouth-disease virus (F2A) owing to its superior ribosome-skipping activity [16]. P2A has also been shown to improve the replication kinetic of Nipah-derived reporter virus [17]. Our goal was to construct reporter DENV2 with very bright fluorescence so that there was a wide separation between the wild-type mean fluorescent intensity and the background signal, giving a wide dynamic range for using the reporter virus to screen for attenuation mutations. We constructed DENV2 reporter viruses expressing fluorescent proteins (FP), namely eGFP (brightness = 34x10^3^ M^-1^cm^-1^) [18] and two bright green fluorescent proteins Clover2 (brightness = 84 x10^3^ M^-1^cm^-1^) [19] and bfloGFP (brightness = 120.9 x10^3^ M^-1^cm^-1^) [20]. The infection of DENV2-eGFP, -Clover2, and–bfloGFP produced fluorescent signals with concentrated signal in the nuclei of Vero, BHK21, and Huh7 cells (Fig 1B). Interestingly, DENV2-bfloGFP produced punctate fluorescent spots that resembled nucleolus (Fig 1B).

Despite no reports of preferred cellular localization of the tested FPs, their fluorescent distributions in the infected cells were varied (Fig 1B), suggesting that the 1x 2A design may produce inconsistent fluorescent signal for different fluorescent proteins. This inconsistency could hamper the application of novel reporters or sensors. Because the FPs were fused to C25 that contains a putative nuclear localization signal (KKAR/K) between amino acids 6–9 [21], we hypothesized that separating C25 from the fluorescent protein gene could fix this problem. Thus, we inserted another 2A sequence from Thoseasigna virus (T2A) in between C25 and the FPs to generate a new set of DENV2-FPs (denoted as 2x 2A in Fig 1A). T2A was chosen for its high ribosome-skipping efficiency and distinct sequence from the downstream P2A [16], allowing for convenient swapping of FP genes by several cloning strategies.

Reporter DENV2 with the FPs separated by two 2A sequences (2x 2A) showed the same whole-cell distribution of fluorescent signal without nuclear concentration in Vero, BHK21, and Huh7 cells (Fig 1C). Similar replication kinetics between the reporter DENV2 with C25-fused (1x 2A) and reporter DENV2 with isolated FPs (2x 2A) were observed (Fig 1D). The replication rates of both types of reporter DENV2 were about an order of magnitude lower than that of the wild-type virus and were delayed by one day (Fig 1D), similar to previous reports of reporter flaviviruses [8,10]. Consistent with their replication kinetics, the foci of the reporter DENV2 were approximately four times smaller than those of wild-type DENV2 (Fig 1E). DENV2-bfloGFP with the 1x 2A design showed heterogeneous foci compared with other DENV2-FPs (Fig 1E). Some of the DENV2-bfloGFP-1x 2A foci were 2–3 times larger than the foci of other DENV2-FPs, which could account for relatively large standard deviation of the measured foci size for this virus (Fig 1E).

**Fig 2:**
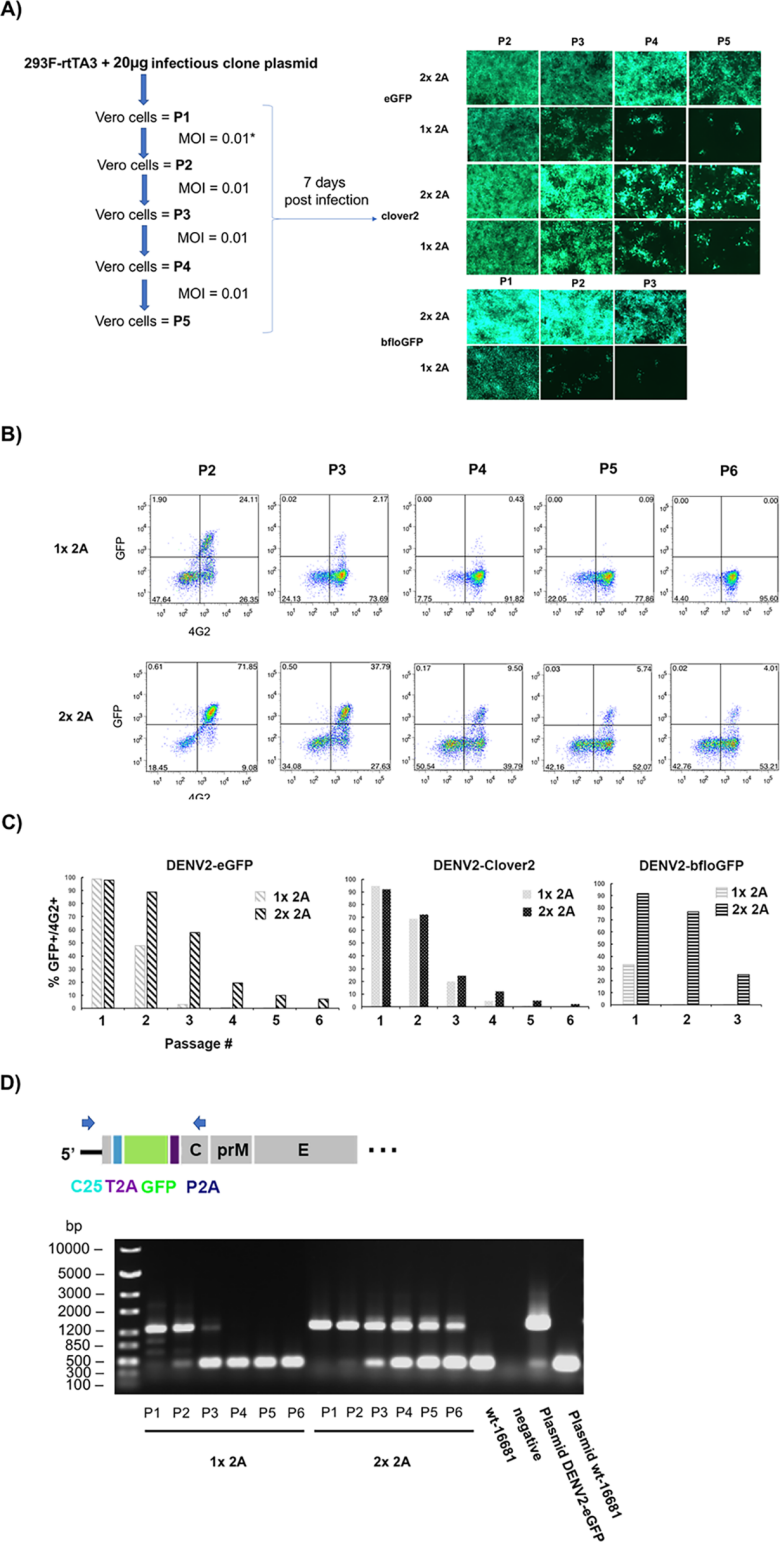
Improved stability of GFP reporter genes on DENV2 genome with 2x 2A design. A) Fluorescent microscopy of infected Vero cells during passaging. The left diagram outlines the passaging of a reporter virus. MOI of 0.01 was used to infect confluent Vero cells for each passage. Green-fluorescent images of infected Vero cells (right panel) were taken seven days post infection. P1, P2, P3, P4, P5, and P6 designate passage numbers. 1x 2A and 2x 2A denote the design of the reporter viruses. B) Flow-cytometry measurements of Vero cells infected with passaged DENV2-eGFP. Confluent Vero cells were infected with viruses from each passage (culture media collected at day 7) at MOI = 0.1. Infected cells were maintained for two days before whole-cell staining with 4G2 mouse antibody and Alexa-647-conjugated anti-mouse antibody. The percentage of each cell population is shown in each quardrant of the scatter plot. The top row shows the measurements of passaged DENV2-eGFP with the 1x 2A design while the bottom row show those of 2x 2A design. C) Quantification of the stability of GFP reporter genes based on flow-cytometry measurements. The stability was measured as the percentage of GFP-positive cells in the populations of infected cells (4G2-positive cells). % GFP/4G2 = (% cells with GFP)/(% cells with 4G2). D) RT-PCR of passaged DENV2-eGFP. Top diagram shows the locations of the primer binding sites (represented by arrows) on the viral genome of reporter DENV2-eGFP. Bottom is the image of agarose gel electrophoresis of the RT-PCR products of viral RNA extracted from passaged viruses.

When the viruses were passaged in Vero cells with multiplicity of infection (MOI) = 0.01 at the start of each round of infection, the fluorescent signal was detectable for 2–3 passages longer for DENV2-FPs with the 2X 2A design than the 1x 2A design (Fig 2A). To test whether the loss of fluorescence was the result of loss of infectivity, we characterized the viruses from each passage by flow cytometry. The medium from infected Vero cells seven days post infection was used to infect Vero cells at MOI = 0.1 so that an infected cell received no more than one virus. The infected Vero cells were then whole-cell stained with anti-envelope 4G2 antibody two days post infection. Analysis of the 4G2-stained cells showed that the loss of fluorescent signal was not the result of loss of infectivity (Fig 2B) as the percentage of 4G2-positive cells (i.e. dengue-infected cells) did not reduce during passaging. Consistent with the results in Fig 2A, reporter viruses with 2x 2A design showed slower drop of the percentage of 4G2-positive cells with GFP signal (%GFP/4G2, Fig 2C). The first passage (P1) stock of DENV2-bfloGFP-1x 2A (Fig 2C, the right bar plot) had less than half of infectivity with fluorescence, suggesting that the large foci observed with this virus (Fig 1E) could be reverted viruses that lost the fluorescent gene. To test whether the loss of fluorescence was due to the deletion of reporter gene from the viral genome, RT-PCR was performed with the extracted viral RNA from the media of the infected Vero cells 7 days post infection. A pair of primers with binding sites in the 5′-UTR and the end of capsid was used to amplify viral cDNA, giving a 1356-bp PCR product from viral cDNA with GFP insert and a 435-bp PCR product from viral cDNA without GFP insert (Fig 2D, upper panel). RT-PCR analysis showed that the loss of fluorescent signal was the result of deletion of inserted GFP genes from the viral genome as the 435-bp PCR product became prominent after the third passage with the concomitant reduction or disappearance of the 1356-bp PCR product (Fig 2D, gel image). Consistent with the results in Fig 2A–2C, the 1356-bp PCR product was observed up to passage 6 for the 2x 2A design while the 1356-bp PCR product was not detectable after passage 3 from the reporter DENV2 with the 1x 2A design (Fig 2D), indicating higher stability of eGFP in the 2x 2A design than the 1x 2A design. Together, these results indicated that the separation of GFPs from C25 and downstream viral genes with 2A sequences increased the stability of inserted GFPs on the genome of DENV2; however, the insert was still not stable in perpetuity.

**Fig 3:**
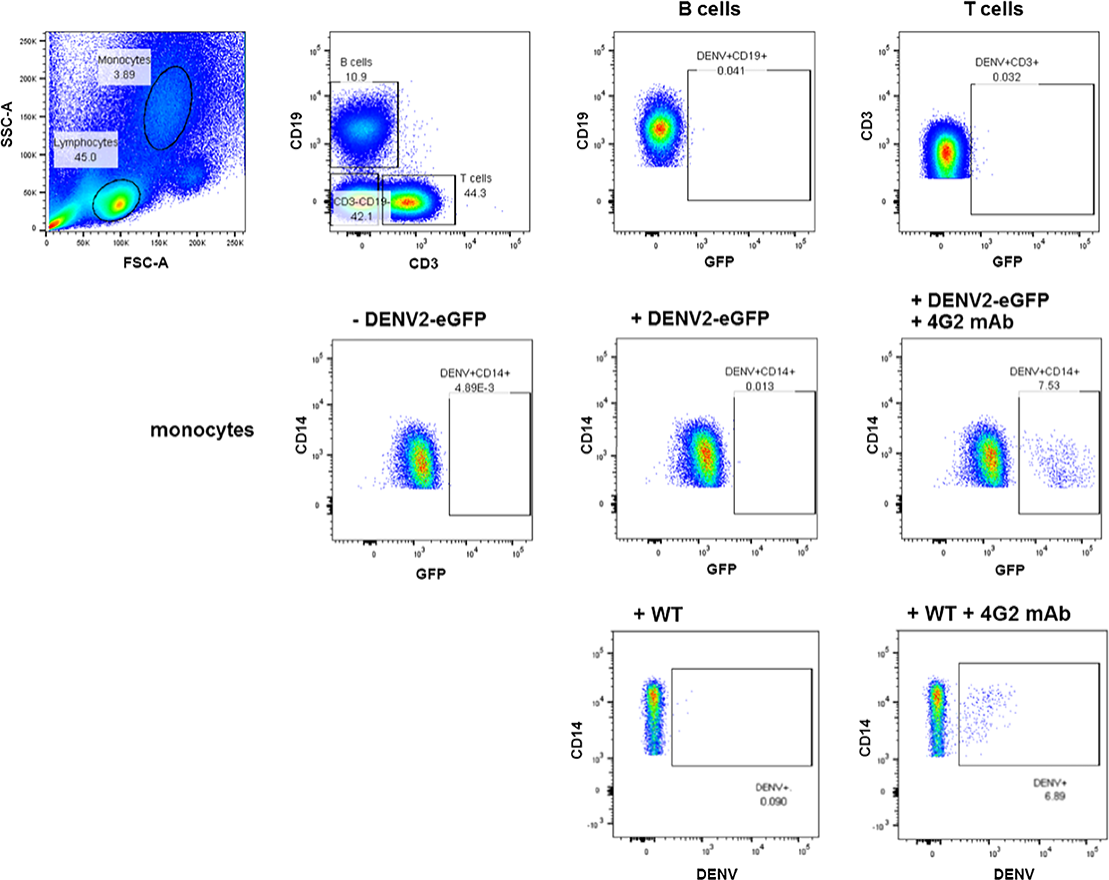
Infectivity of DENV2-eGFP with the 2x 2A design in human peripheral blood mononuclear cells (PBMC). PBMCs were infected with DENV2-eGFP in the presence of 4G2 antibody (1 μg/ml). Top row: Monocytes and lymphocytes were gated based on size (FSC-A) and granularity (SSC-A) (far left). The lymphocyte population was then divided into B cells (CD19+CD3-) and T cells (CD19-CD3+). Middle row: Frequency of DENV2-eGFP positive cells in monocyte (CD14+) population (mock infection, left; infection with virus but no antibody, middle; infection with virus and antibody (ADE), right). DENV2-eGFP positive cells in individual populations are boxed in each plot. Bottom row: Frequency of DENV2-positive monocytes infected with wild-type DENV2-16681 (left; infection with virus but no antibody, right; infection with virus and antibody (ADE), right).

**Fig 4:**
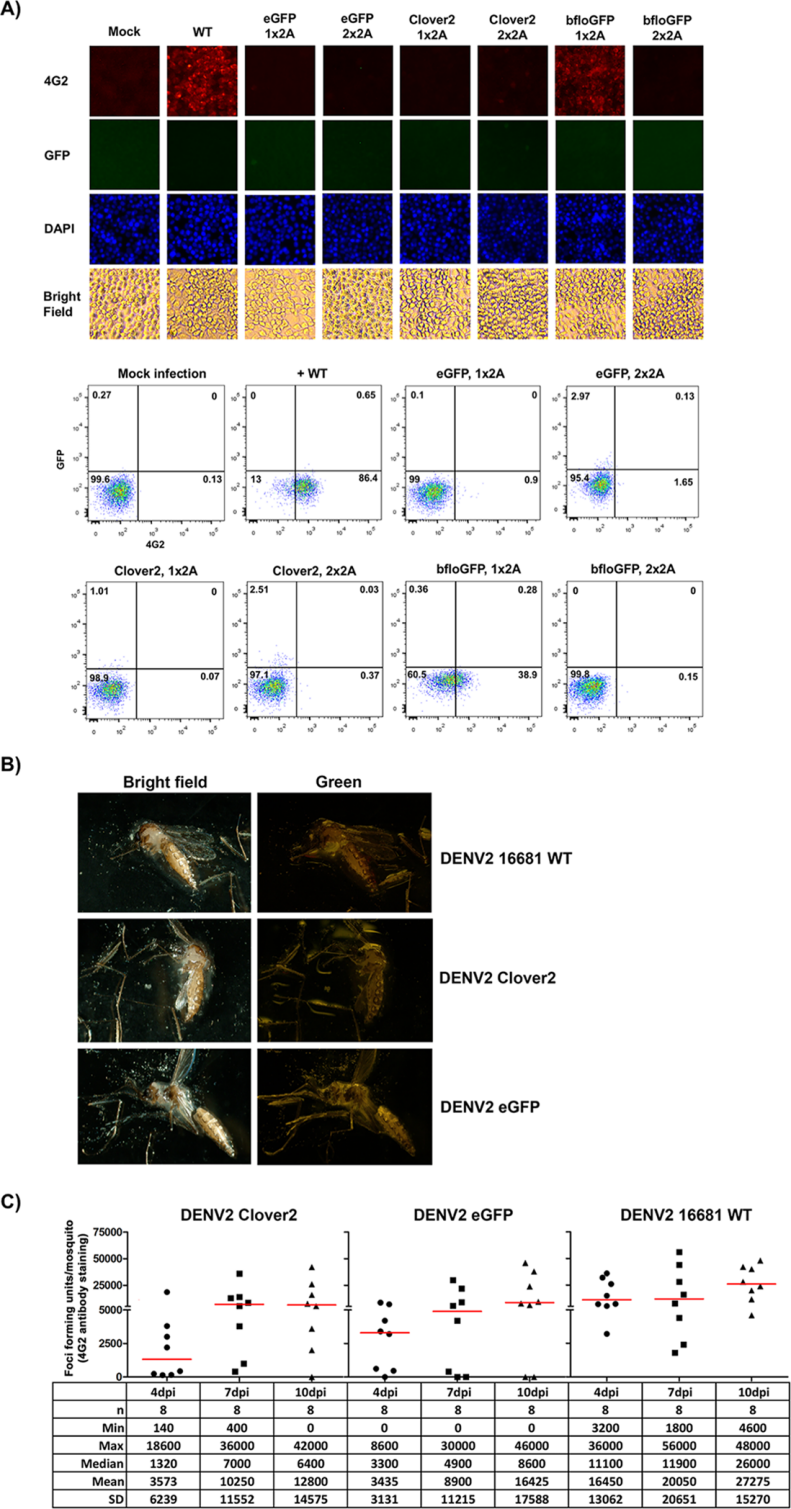
Inability of the reporter DENV2 to replicate in mosquito host. A) Fluorescent microscopy (top panel) and flow cytometry (bottom panel) of C6/36 cells infected with DENV2-16681 (WT) and DENV2-GFPs with 1x 2A and 2x 2A designs. C6/36 cells were infected with a DENV2 virus at MOI = 0.1 and cultured for 3 days before analyses. Infected cells were identified by immunostaining with 4G2 antibody. The number in each quadrant of the flow cytometry scatter plot represents the percentage of cells in the quadrant. B) Fluorescent microscopy of *Aedes aegypti* mosquitoes that received DENV2-16681 (WT), DENV2-Clover2-2x 2A, and DENV2-eGFP-2x 2A by intrathoracic injection. The mosquitoes were imaged seven days after injection. C) Propagation of viruses in the mosquitoes injected with DENV2-16681 (WT), DENV2-Clover2-2x 2A, and DENV2-eGFP-2x 2A. The infectious titer (FFU/mosquito) was quantified from the whole-body homogenate of a mosquito by immunostaining foci assay at 4, 7, and 10 days post intrathoracic injection (dpi). The table underneath the plot reports the descriptive statistics of the infectious-titer measurements that include the number of the mosquitoes (n), the minimum (Min), the maximum (Max), the median, the mean, and the standard deviation (SD) of the infectious titers for each condition.

To test the utility of the reporter viruses in infection analysis in natural target cells, we tested their infectivity in target primary human cells. Human peripheral blood mononuclear cells (PBMC) were infected by wild-type DENV2 and DENV2-eGFP with the 2x 2A design at MOI of 0.5 with or without 4G2 antibody. Green fluorescence could be detected by flow cytometry only in the CD14+ monocytes of PBMC infected with DENV2-eGFP in the presence of 4G2 antibody, but not in B cells, T cells, and NK cells 3 days post infection (Fig 3). The infection rates of DENV2-eGFP (7.53%) and wild-type DENV2 (6.89%) in CD14+ monocytes with 1 μg/ml 4G2 antibody were similar (Fig 3), indicating equivalent infectivity in CD14+ monocytes between the two viruses. The observed antibody-dependent enhancement (ADE) of infections in CD14+ was consistent with previous reports of *in vitro* infection in human PBMC with wild-type dengue viruses [22,23]. Although all the fluorescent reporter DENV2 could replicate in all the tested mammalian cells, they failed to replicate in mosquito cell line C6/36 (Fig 4). In contrast to wild-type DENV2-16681, immunofluorescent assay and analysis by flow cytometry detected background levels of 4G2-positive cells in C6/36 cells infected with P1 stock of DENV2-GFPs (both 1x and 2x 2A designs) at MOI ~ 0.3, except for DENV2-bfloGFP with 1x 2A design which produced 38% of 4G2-positive, GFP-negative (4G2+/GFP-) cells (Fig 4A). Detection of 4G2+/GFP- cells in C6/36 infected with DENV2-bfloGFP-1x 2A was congruent with the presence of a significant amount of reverted virus in the P1 stock (Figs 1E and 4C, bfloGFP 1x2A). Consistent with the results in C6/36 cells, intrathoracic injection of DENV2-Clover2 and DENV2-eGFP with the 2x 2A design into *Ae*. *aegypti* mosquitoes produced neither green fluorescence in the mosquitoes (Fig 4B) nor detectable GFP+/4G2+ foci in the homogenized whole mosquito bodies. Nevertheless, the GFP-/4G2+ foci could be detected in the homogenized whole mosquito bodies (Fig 4C). The delay in the rise of detectable titers in the mosquitoes injected with DENV2-Clover2 and DENV2-eGFP compared with those of wild-type DENV2 (Fig 4C) was consistent with the fact that the GFP-/4G2+ foci in homogenates of mosquitoes infected with both reporter viruses could be derived from the small amount of non-fluorescent DENV2 (~ 10%) in the P1 stock of the reporter virus (Fig 2C).

Multi-color fluorescent proteins enable tagging viruses with different colors to visualize their infections and interactions. Different colors can be used to designate genetic variants and timing of infection. By tagging different genetic variants with different colors, interactions among the variants and interference may be analyzed. By tagging viruses of the same viral genetic background according to its timing of infection, multi-color reporter viruses can be used to analyze the superinfection exclusion phenomenon [24,25].

**Fig 5:**
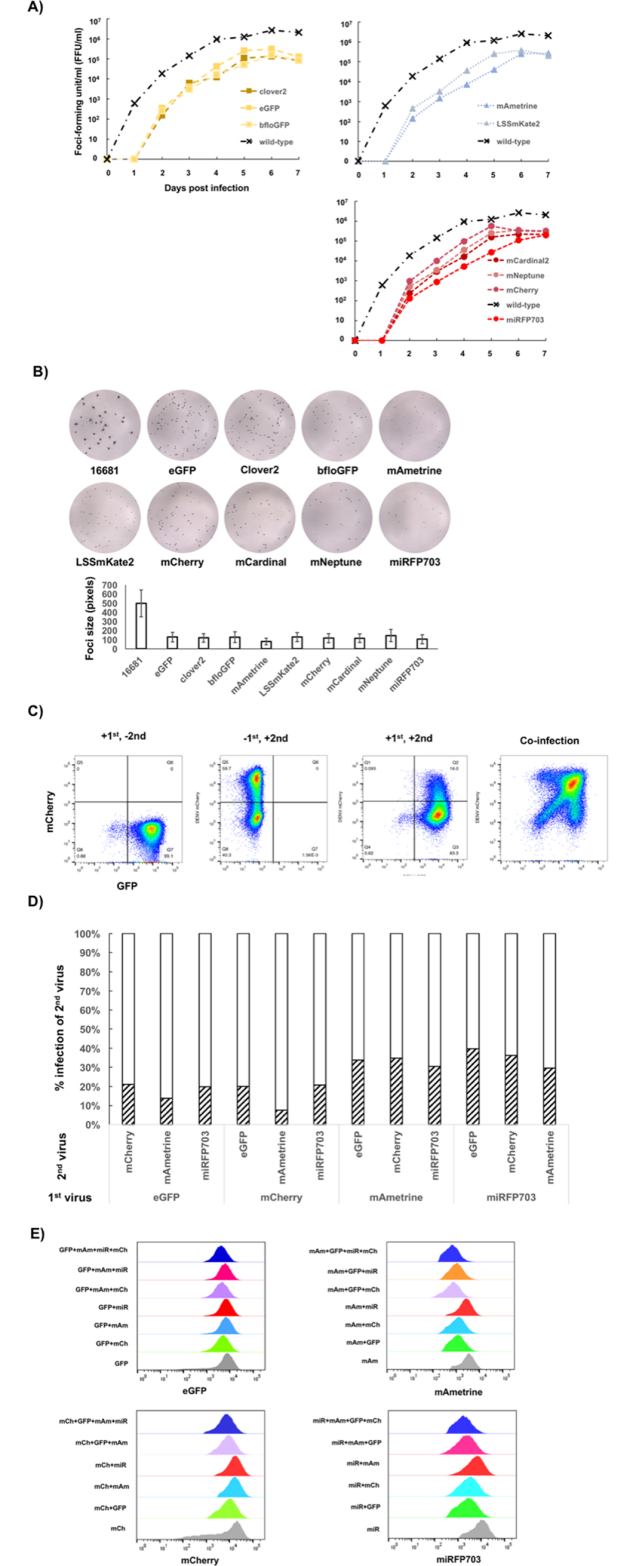
A panel of multi-color fluorescent DENV2 with comparable replicative abilities. A) Multi-step replication kinetics of multi-color fluorescent DENV2. Confluent Vero cells were infected with one virus at MOI = 0.01. The culture media was sampled from the flask every 24 hours. Infectious virus titer in the media was assayed by foci-forming assay. B) Focus images of the wild-type (16681) and the multi-color DENV2 (top panel) and the bar graph comparing the sizes of their foci (bottom panel). Focus size was quantified by the number of pixels that it occupied on a digitized image taken on ELISpot reader. Each bar represents the average of the measured focus sizes and the error bars represent standard deviation. Between 143 and 713 foci were used for focus size quantification. C) Representative scatter plots of K562-CD209 infected with DENV2-eGFP and DENV2-mCherry. The first (with DENV2-eGFP) and the second (with DENV2-mCherry) infections were 24 hours apart. The infected cells were analyzed 3 days post infection. In the case of co-infection (right plot), the cells were infected by both viruses at the same time and analyzed 2 days post infection. D) Comparison of infection percentages of the second virus in K562-CD209 between the presence (diagonal-stripe bars) and the absence (empty-bar) of the first virus infections. The infection percentages in the absence of the first virus infections are normalized to 100% for comparing the extent of reduction (or exclusion) conferred by the first virus. E) Histograms of fluorescent intensities for each fluorescent reporter measured from populations of K562-CD209 infected with single virus, and co-infected with two, three, and four reporter DENV2 of different colors.

Having shown the improved stability and fluorescent distribution of the 2x 2A design, we tested the expression of other fluorescent proteins with this design. We used the Cas9-gRNA system [26] to excise Clover2 gene and generate the infectious-clone backbone for Gibson assembly with PCR products of other fluorescent reporter genes. The Cas9-gRNA system enabled one-step cloning to exchange the reporter gene without the need to go through intermediate constructs or to modify the original template sequence to accommodate the swapping of FP genes. We constructed fluorescent DENV2-16681 with mCherry [27], mAmetrine [28], miRFP703 [29], LSSmKate2 [30], mCardinal [31], and mNeptune2 [31]. Multi-step replication kinetics showed that the fluorescent reporter DENV2 yielded an approximate of one-log lower infectious titers compared with the wild-type 16681 virus between day 6–7 (Fig 5A). Whereas most of the fluorescent reporter DENV2 showed similar kinetics, DENV2 with miRFP703 and LSSmKate2 showed slower replication kinetics than the others, with an approximately two-log reduction compared with the wild-type between days 2–5 post infection (Fig 5A). The foci of these two reporter viruses had similar sizes to all other fluorescent reporter DENV2 (Fig 5B).

Two additional tests were performed to gauge the relative replicative abilities of the viruses. First, two multi-color DENV2 were used to infect cells one day apart from each other to measure the ability of the first virus to exclude the infection of the second virus, a phenomenon known as superinfection exclusion. Second, combinations of multi-color DENV2 were used to co-infect cells at the same time to assess the potential fitness advantage of one virus in the presence of other virus in the same infected cell.

We tested superinfection exclusion by infecting multi-color reporter DENV2 in K562 cells with constitutive expression of a dengue receptor CD209 (DC-SIGN) (K562-CD209). The cells were first infected with a fluorescent DENV2 and then infected with the second reporter DENV2 of different color 24-hour post first infection. The fluorescent measurement was done 72 hours after the first infection by flow cytometry (K562-CD209, Fig 5C). Superinfection exclusion by the first virus was measured as the reduction of the infection percentage of the second virus relative to the infection percentage (normalized to 100%) of a control where the second virus was infected without the first virus. Analysis of superinfection exclusion showed that DENV2-eGFP, mCherry, mAmetrine, and miRFP703 in K562-CD209 could exclude the second virus to a similar extent (Fig 5D). These results indicated that fluorescent tags have little influence on the superinfection exclusion.

To compare relative replicative abilities of multi-color DENV2 during co-infection, combinations of two, three, and four orthogonal fluorescent reporter DENV2 (eGFP, mCherry, mAmetrine, miRFP703) were co-infected in K562-CD209 with MOI in the range of 0.5–2.0. The fluorescent measurement by flow cytometry was done 48 hours post infection. The fluorescent intensity for each reporter protein was measured in populations of co-infected cells and plotted as histograms. For each fluorescent protein, the comparison of fluorescent histograms measured from cell populations infected with one, two, three, and four viruses showed significant overlaps (Fig 5C and 5E), indicating that there were no replication advantages conferred by different fluorescent reporter genes in the co-infection setting.

## Discussion

Here we showed that flanking both sides of the GFP reporter gene inserted after C25 with 2A ribosome-skipping sequences could improve the stability of the reporter gene and enabled the construction of a set of multi-color reporter DENV2 (Figs 1, 2 and 5). With the diversity of amendable fluorescent proteins and the infectivity of the reporter viruses in natural target hosts such as human CD14+ monocytes (Fig 3), this design should be able to support other reporter genes such as luciferase-based reporters for sensitive imaging in animal models and other biosensors to characterize the state of infected cells.

It is not yet clear why the separation of GFP reporter gene from C25 sequence increased the stability of the reporter gene in the viral genome (Fig 2). Having C25 sequence fused to the N-terminus of a reporter protein could affect how the reporter functions in the cells. The altered behavior of C25-bfloGFP was particularly striking with its punctate localization compared with C25-eGFP and C25-clover2 (Fig 1). The degree of altered GFP localizations coincided with the degree of instability of the reporter viruses (Fig 2). It is not yet clear how the insertion of GFPs made DENV2 incapable of replication in the mosquito hosts (Fig 4). The inserted reporter gene could disturb the nearby RNA structures that contributed to the cyclization and replication of viral genome, reducing the fitness of reporter virus [32]. The disruption may affect viral replication in mosquito host cells as our reporter viruses could not infect cultured mosquito cells and live mosquitoes by intrathoracic injections (Fig 4). The increased size of reporter viral genome could also decrease the efficiency of virus assembly and its fitness.

The analyses of multi-color DENV2 infections (Fig 5) showed that the 2x 2A design could be used to generate a panel of multi-color viruses with comparable replicative abilities. The results indicated that the fluorescent protein tags had little influence during multi-virus infection experiments. Thus, it should be possible to apply this reporter system to investigate interactions among different virus strains or genetic variants.

The stability of reporter gene in the viral genome is a key factor for certain studies. Application of reporter virus to analyze persistent infections and transmission in animal models will need stable reporter gene expression that is maintained through numerous round of viral replication during these processes. The loss of reporter gene in the middle of the experiment such as high-throughput screening by fluorescent-activated cell sorting could introduce noise into single-cell measurements of virus replication. The 2x 2A design could improve our abilities to do these experiments as demonstrated by slower loss of GFPs than 1x 2A designs (Fig 2).

It should be possible to adapt the 2x 2A design to generate reporter DENV1-4 with different color tags. The multi-color reporter viruses could be used to dissect the process of co-infection of DENV1-4 in animal models by imaging and single-cell analysis. The information gained from such studies could be particularly useful for understanding the interference phenomenon that is relevant to the development of combined live-attenuated dengue vaccine to confer protective immunity against all serotypes. Despite the availability of licensed dengue vaccine, effective protection against all four serotypes is still an issue that needs to be addressed [33,34].

## Materials and methods

### Cell lines

Vero, BHK21, and 293F-rtTA3 cells were maintained in DMEM supplemented with 10% heat-inactivated fetal calf serum (HI-FBS, Invitrogen), 1x penicillin/streptomycin (Invitrogen), 1x Glutamax (Invitrogen), 1mM sodium pyruvate (Sigma), 1x MEM nonessential amino-acids (Invitrogen), and 4.5g/L glucose (D10). K562 and K562-CD209 were maintained in RPMI1640 (HyClone^TM^) supplemented with 10% HI-FBS, 1x penicillin/streptomycin, and 1x Glutamax (R10). Huh7 was maintained in R10 supplemented with 1x MEM nonessential amino-acids. Both K562-CD209 and 293F-rtTA3 were generated by lentivirus transduction. A cDNA clone of human CD209 (DC-SIGN) was sub-cloned into lentiviral plasmid pLenti-SFFV-NEO, constructed by replacing the CMVtight promoter on pLenti-CMVtight-NEO [35] with the SFFV promoter. The open-reading frame of transcription activator rtTA3 was sub-cloned into pLenti-CMVtight-Blast [35]. Each lentiviral plasmid was co-transfected with packaging plasmids (pCMV-VSV-G (Addgene #8454) and pSPAX2 (Addgene #12260)) into 293T cells to generate lentiviral particles. K562 cells were transduced with lentiviral particles for CD209 using 8 μg/ml polybrene in R10 media. A single, stable clone of K562-CD209 cells was selected with 250 μg/ml G418 (Invivogen) and isolated by limiting dilution. 293F cells were transduced with lentiviral particles for rtTA3 using 8 μg/ml polybrene (Sigma). Transduced cells were selected by 10 μg/ml blasticidin (Invivogen) and used as a pool of surviving cells. All the mammalian cell lines were cultured at 37°C, 5% CO_2_, and 80% humidity. *Aedes albopictus* C6/36 cells were maintained in L15 (HyClone^TM^) supplemented with 10% tryptose phosphate broth (Sigma), 10% heat-inactivated fetal-calf serum (HI-FBS, Invitrogen), 1x penicillin/streptomycin (Invitrogen), 1x Glutamax (Invitrogen). C6/36 cells were cultured at 28°C.

### Construction of virus infectious clones

All the infectious-clone plasmids of the viruses used in this work were generated by Gibson assembly of the linear double-stranded DNA derived from either PCR, Cas9-cleaved plasmids, or synthetic linear DNA. A PCR amplification was carried out with Phusion DNA polymerase (NEB). Asembly reactions were transformed into *Escherichia coli* DH5alpha and plated onto LB-agar with 50 μg/ml amplicillin. Culture plates were incubated at 22°C for 2 ½ days. Colonies were picked and grown in LB broth with 25 μg/ml amplicillin at 22°C. Plasmid DNA was prepared using either Qiagen miniprep or midiprep kits. All the plasmid clones were verified by Sanger DNA sequencing. The primers used for cloning are reported in Table 1.

**Table 1:** Sequences of primers and protospacer sequence of gRNAs used in this work.

Name	Sequence (5' - 3')
pUC57-T7-prom-fw	GATCCCTAATACGACTCACTATA
pUC57-sgRNA-rv	AAAAAAAGCACCGACTCG
C-dv2-16681-start-fw	CAATATGCTGAAACGCGAGAGAAAC
P2A-rv	AGGTCCAGGGTTCTCCTCCACG
hCMV-DV216681+8-fw-Gib	AGAGCTCGTTTAGTGAACCGAGTTGTTAGTCTACGTGGACCGACAAAGACAG
C-dv2-16681-start-rv	GTTTCTCTCGCGTTTCAGCATATTG
P2A-C-16681-fw	CGTGGAGGAGAACCCTGGACCTATGAATAACCAACGGAAAAAGGCG
E-dv2-16681-end-rv	CCTGCACCATGACTCCCAAATAC
E-dv2-16681-end-fw	GTATTTGGGAGTCATGGTGCAGG
6685_6709_rv_den2	CCAGTATTATTGAAGCTGCTATCCA
DV2-16681-6695_6718-fw	CTTCAATAATACTGGAGTTTTTTCTCATAG
3’ UTR-DV2	AGAACCTGTTGATTCAACAGCACCATTCCATTTTCTGGCGTTCTGTGCCTGGAATGAT
HDV-fw-dv2	CTGTTGAATCAACAGGTTCTGGGTCGGCATGGCATCTCC
CMV-5’ UTR-DV2	CGGTTCACTAAACGAGCTCTGCTTATATAGACCTCCCACCG
C25-GFP-fw	CGAGAGAAACCGCGTGTCGACTATGGTGAGCAAGGGCGAGGAG
P2A-GFP-rv	CAGGCTGAAGTTAGTAGCTCCGCTTCCCTTGTACAGCTCGTCCATGCCG
pUC57-T7-prom-fw	GATCCCTAATACGACTCACTATA
and pUC57-sgRNA-rv	AAAAAAAGCACCGACTCG
C25-bfloGFPa1-fw	CGAGAGAAACCGCGTGTCGACTATGCCTCTGCCCGCAACCCACG
P2A-bfloGFPa1-rv	CAGGCTGAAGTTAGTAGCTCCGCTTCCATGGTGATGGTGATGGTGAGCC
C25-GFP-fw	CGAGAGAAACCGCGTGTCGACTATGGTGAGCAAGGGCGAGGAG
P2A-GFP-rv	CAGGCTGAAGTTAGTAGCTCCGCTTCCCTTGTACAGCTCGTCCATGCCG
NS4B-16681-end-fw	GAAGAACACAACCAACACAAGAAGG
C25-16681-rv	AGTCGACACGCGGTTTCTCTCGC
C25-C26-16681-fw	GAGAAACCGCGTGTCGACTGTGCAACAGCTGACAAAGAGATTCTCAC
NS4B-16681-end-rv	CCTTCTTGTGTTGGTTGTGTTCTTC
T2A-clover-fw	GGAAGCGGAGAGGGCAGAGGAAGTCTGCTAACATGCGGTGACGTCGAGGAGAATCCTGGACCTATGGTGAGCAAGGGCGA GGAGCTGTTCA
T2A-C25-rv	AGGTCCAGGATTCTCCTCGACGTCACCGCATGTTAGCAGACTTCCTCTGCCCTCTCCGCTTCCAGTCGACACGCGGTTTC TCTCGCGTTTC
T2A-GFP-fw	GACGTCGAGGAGAATCCTGGACCTATGGTGAGCAAGGGCGAGGAG
P2A-GFP-rv	CAGGCTGAAGTTAGTAGCTCCGCTTCCCTTGTACAGCTCGTCCATGCCG
T2A-bfloGFPa1-fw	TGCGGTGACGTCGAGGAGAATCCTGGACCTATGCCTCTGCCCGCAACCCACG
P2A-bfloGFPa1-rv	CAGGCTGAAGTTAGTAGCTCCGCTTCCAGCCAGCTCGTAGAACGCCTTCTC
T2A-mTSaph-tdT-mAmet-fw	TGCGGTGACGTCGAGGAGAATCCTGGACCTATGGTGAGCAAGGGCGAGGAG
P2A-mCardinal-mNeptune2-rv	CAGGCTGAAGTTAGTAGCTCCGCTTCCCTTGTACAGCTCGTCCATGCCATT
T2A-mNeptune2-fw	TGCGGTGACGTCGAGGAGAATCCTGGACCTATGGTGTCTAAGGGCGAAGAGCTGA
P2A-mCardinal-mNeptune2-rv	CAGGCTGAAGTTAGTAGCTCCGCTTCCCTTGTACAGCTCGTCCATGCCATT
P2A-tdTomato-mAmet-rv	CAGGCTGAAGTTAGTAGCTCCGCTTCCCTTGTACAGCTCGTCCATGCCGT
T2A-LSS-mKate2-fw	TGCGGTGACGTCGAGGAGAATCCTGGACCTATGAGCGAGCTGATTAAGGAGAACATG
P2A-LSSmKate2-rv	CAGGCTGAAGTTAGTAGCTCCGCTTCCATTAAGCTTGTGCCCCAGTTTGCTAGG
T2A-miRFP703-fw	TGCGGTGACGTCGAGGAGAATCCTGGACCTATGGTAGCAGGTCATGCCTCTGGC
P2A-miRFP703-rv	CAGGCTGAAGTTAGTAGCTCCGCTTCCGCTCTCAAGCGCGGTGATCCG
5UTR-DV2-16681-fw	AGTTGTTAGTCTACGTGGACCGACAAAG
C-end-16681-rv	CGCCATCACTGTTGGAATCAGCATAATG
gRNA-C25-16681	GAGAAACCGCGTGTCGACTA
gRNA-eGFP-P2A	GACGAGCTGTACAAGGGAAG
gRNA-T2A-Clover2	ATGGTGAGCAAGGGCGAGGA
gRNA-Clover-eGFP-P2A	CTTCCCTTGTACAGCTCGTC

To modify the infectious-clone plasmids of DENV2 to generate reporter-virus constructs reported here, Cas9 and guide RNA (gRNA) were employed to make specific cuts on the plasmids. Cas9 was obtained from pMJ806 and purified with NiNTA (Qiagen) and HiTrap SP (GE Healthcare) according to the reported protocols [26]. Guide RNA was produced by *in vitro* transcription with T7 RNA polymerase using PCR products of the gRNA with T7 promoter sequence (primers pUC57-T7-prom-fw and pUC57-sgRNA-rv). The cleavage reaction was set up based on the condition reported by Jinek et al. (2012) [26]. The sequences of protospacers of gRNA are reported in Table 1.

#### Construction of DENV2-eGFP, bfloGFP, clover2 1x2A

pGEM-TRE-DENV2-eGFP-1x2A was constructed by Gibson assembly of one PCR product for C25-eGFP-P2A (C-dv2-16681-start-fw & P2A-rv) and four PCR products from DENV2-16681 cDNA: three amplified from cDNA of DENV2-16681 (hCMV-DV216681+8-fw-Gib & C-dv2-16681-start-rv; P2A-C-16681-fw &E-dv2-16681-end-rv; E-dv2-16681-end-fw & 6685_6709_rv_den2; DV2-16681-6695_6718-fw & 3’ UTR-DV2) and one amplified from pGEM-TRE-HDV plasmid (HDV-fw-dv2 & CMV-5’ UTR-rv) [36]. pGEM-TRE-HDV was derived from low-copy plasmid pGEM-T-Easy (Promega). The genomic sequence of dengue virus was under the control of an inducible promoter with tetracycline response element (TRE) and included the hepatitis delta virus ribozyme on the 3’ end. The infectious-clone plasmids of DENV2-bfloGFP-1x 2A and DENV2-clover2-1x 2A were constructed by Gibson assembly of the PCR products of bfloGFP (amplified by C25-bfloGFPa1-fw & P2A-bfloGFPa1-rv) and Clover2 (amplified by C25-GFP-fw & P2A-GFP-rv), respectively, with eGFP-deleted backbone of pGEM-TRE-DENV2-eGFP-1x2A excised with Cas9, gRNA-C25-16681, and gRNA-eGFP-P2A.

#### Construction of infectious-clone plasmid of DENV2-16681

The infectious-clone plasmid of the wild-type virus (without reporter gene and 2A sequence) was constructed by Gibson assembly of two PCR products amplified with primers NS4B-16681-end-fw & C25-16681-rv and C25-C26-16681-fw & NS4B-16681-end-rv using pGEM-TRE-DENV2-eGFP-1x2A as the template. The infectious clone plasmid was constructed by Gibson assembly of the PCR products of the pGEM-TRE-HDV vector and two PCR products amplified from DENV2-16681 cDNA.

#### Construction of DENV2- eGFP, bfloGFP, clover2 2x2A

pGEM-TRE-DENV2-clover2-1x 2A was cleaved with Cas9 using the gRNA that targeted the junction between C25 and N-terminus of clover2. The annealed oligos (T2A-clover-fw and T2A-C25-rv) were joined with the Cas9-cleaved pGEM-TRE-clover2-1x 2A to incorporate T2A sequence between C25 and clover2, generating the infectious-clone plasmid of DENV2-Clover2-2x 2A. The infectious-clone plasmids of DENV2-eGFP-2x2A and DENV2-bfloGFP-2x2A were generated by replacing Clover2 on the infectious-clone plasmid of DENV2-Clover2-2x 2A with eGFP and bfloGFP, respectively. The infectious-clone plasmid of DENV2-Clover2-2x 2A was cleaved with Cas9 and two gRNAs (gRNA-T2A-Clover2 and gRNA-Clover-eGFP-P2A) to excise Clover2, generating a backbone for inserting other fluorescent reporter genes. The PCR products of bfloGFP and eGFP for cloning were amplified with T2A-GFP-fw & P2A-GFP-rv and T2A-bfloGFPa1-fw & P2A-bfloGFPa1-rv, respectively. The infectious-clone plasmids for DENV2-mCardinal2, -mCherry, -mNeptune, -mAmetrine, LSSmKate2, and -miRFP703 were also generated by this method using the following pairs of primers to amplify the fluorescent reporter genes for insertion: T2A-mTSaph-tdT-mAmet-fw & P2A-mCardinal-mNeptune2-rv, T2A-GFP-fw & P2A-GFP-rv, T2A-mNeptune2-fw & P2A-mCardinal-mNeptune2-rv, T2A-mTSaph-tdT-mAmet-fw & P2A-tdTomato-mAmet-rv, T2A-LSS-mKate2-fw & P2A-LSSmKate2-rv, T2A-miRFP703-fw & P2A-miRFP703-rv, respectively.

### Virus production and titration

20 μg of plasmid prepared by Qiagen midiprep kit was transfected into 2.5 x10^6^ cells of 293F-rtTA3 on 100-mm tissue-culture treated dish using PEI. The cells were maintained in 10 ml ISF1 media (Biochrom) supplemented with 1 μg/ml doxycycline one day after transfection. The media was exchanged daily. The media containing virus particles was harvested 3 days after transfection and clarified by 9,100 xg centrifugation at 4°C for 10 minutes. The clarified medium was then used to infect confluent Vero cells in a T75 flask overnight. The infection medium was removed the next day and replaced fresh ISF1 medium. Media were replaced every two days. Media containing virus particles were harvested between 4–10 days post infection. The harvested media was clarified by 9,100 xg centrifugation at 4°C for 5 minutes. The supernatant was then supplemented with HI-FBS (final concentration of 20%), aliquoted, and stored frozen at -70°C until use. The resulting virus stocks were designated as passage-1 virus (P1). Concentrated stocks required for human PBMC were obtained from the clarified supernatant by membrane-filtration and centrifugation method with 50-kDa or 100-kDa cutoff devices. Concentrated stocks of DENV2-eGFP-2x 2A and DENV2-clover2-2x 2A with infectious titers of ~ 5x10^6^ FFU/ml and ~ 1x10^7^ FFU/ml were generated, respectively.

Virus stock titer were quantitated by foci assay. Vero cells were seeded at ~50,000 cells/well in 96-well plate one day prior to the infection. A virus sample/stock was 10-fold serially diluted in cold MEM2. 50 μl samples of the dilutions were added to each well in duplicate. After two-hour incubation with virus dilutions at 37°C, 5% CO_2_, and 80% humidity, each well was overlaid with 150 μl MEM2 supplemented with 1.2% carboxymethylcellulose (Sigma). The plate was incubated at 37°C, 5% CO_2_, and 80% humidity for three days. The cells were fixed with 3.7% formaldehyde in 1xPBS and permeabilized with 2% TritonX-100 in 1xPBS. Staining was performed with 4G2 as the primary antibody, horseradish peroxidase (HRP)-conjugated anti-mouse IgG (Dako) as the secondary antibody and DAB (3,3’-diaminobenzidine, Sigma) as the chromogenic substrate.

### Foci quantification

Dried, stained virus titer plates were scanned and digitized with KS ELISPOT reader (CarlZeiss). Foci in each well image were characterized in ImageJ [37]. An image of each well was extracted from the scanned image using a circular mask (diameter of 850 pixels). Well images were band-pass filtered (large structures down to 40 pixels, small structures up to 3 pixels) and binarized to separate foci and background. Then, the areas of binarized foci were quantified (the number of pixels) using the “Analyze Particles” function.

### Multi-step replication kinetic

Vero cells were plated at a density of 1x10^6^ cells/T25 flask in MEM10 medium one day prior to infection. The cells were counted and infected by replacing the medium with the infection media and incubated at 37°C, 5% CO_2_, and 80% humidity for 2 hours. The infection media were prepared by mixing a virus with MEM medium supplemented with 2% HI-FBS and 1xPen/StrepG (MEM2) in a total volume of 2 ml so that an MOI = 0.01 was obtained. The infected cells were washed three times with 5 ml PBS and replenished with 5.5 ml MEM2. 500 μl of the media was collected as the day-0 sample and cell culture continued at 37°C, 5% CO_2_, and 80% humidity. 500 μl samples of the media was collected every 24-hour and the cell culture volume adjusted with the same amount of MEM2. The collected media samples were clarified by centrifugation at 4,000 rpm, 4°C, 15 minutes. 400 μl of the clarified supernatant was mixed with 100 μl HI-FBS, aliquoted, and stored in -70°C until titration by foci assay.

### Passaging of reporter DENV2

Vero cells were plated at the density of 1.5x10^6^ cells/T25 flask in MEM10. One day after seeding, the cells were counted and infected with the virus at MOI = 0.01 in 5 ml ISF1 media and then kept at 37°C, 5% CO_2_, 80% humidity. The media were replenished with 5 ml ISF1 every two days. The passaged virus in the media was harvested seven days post infection and clarified by centrifugation at 5,800 xg, 4°C, 5 minutes. The clear supernatant was supplemented with 20% HI-FBS and stored at -70°C. The infectious titer of the frozen virus was quantitated by foci assay. The frozen virus was then used to start a new round of the passaging by repeating the infection in fresh Vero cells as detailed above.

The virus collected from each passage was used for analysis of stability by flow cytometry (Fig 2B). Vero cells were infected with the virus at MOI = 0.1. The cells were cultured for 2 days before harvesting for co-staining analysis with 4G2 mAb. Detached infected Vero cells were fixed and permeabilized with 3.7% formaldehyde and 0.5% saponin, respectively. Permeabilized cells were stained with 4G2 mAb at 4°C for 1 hour, followed by two washes with 0.5% saponin in 1X PBS and incubation with goat anti-mouse IgG conjugated with Alexa Fluor 647 (Invitrogen) at 4°C for 30 minutes. Stained cells were analyzed on BD LSR Fortessa. Data analysis was performed using FlowJo software version 10.1.

### RT-PCR

Viral RNA was extracted from the passaged virus (media collected 7-day post infection in Vero cells) using Viral QIAmp kit (Qiagen) following the manufacturer’s protocol. The viral RNA was converted into cDNA using SuperScriptIII kit (Invitrogen) with random hexamers following the manufacturer’s protocol. The cDNA was used as the template for RT-PCR using primers 5UTR-DV2-16681-fw and C-end-16681-rv. The PCR amplification was performed with Phusion DNA polymerase with 3% DMSO in 1X HF buffer. The amplification started with 98°C for 3 minutes followed by 25 cycles of 98°C for 15 seconds, 72°C for 10 seconds, and 72°C for 1 minute.

### Infections with multi-color DENV2

For super-infection exclusion, K562-CD209 cells was infected with the first virus by adding 250 μl of a P1 virus stock to a 600 μl suspension of 200,000 cells in R10. The mixture was then incubated for 24 hours at 37°C, 5% CO_2_, and 80% humidity. To infect the cells with the second virus, 600 μl of the cultured media was drawn out and 250 μl of a P1 stock of the second virus and 450 μl of R10 media were added back. The mixture of the second virus and the cells was incubated for another 24 hours in the same condition as the first infection. For the null infections, 250 μl ISF1 with 20% HI-FBS was used in place of a virus stock for both the first and the second infections. The cells were harvested and kept on ice for fluorescent measurement by flow cytometry. Properties of fluorescent proteins, laser and detection filter used in flow cytometer are shown in Table 2.

**Table 2:** Properties of fluorescent proteins, laser and detection filter in flow cytometer.

Fluorescent protein	Ex max (nm)	Em max (nm)	Laser	Detection filter (nm)
eGFP	488	507	Blue (488 nm)	530/30
mCherry	587	610	Yellow-green (561 nm)	610/20
miRFP703	674	703	Red (640 nm)	730/45
mAmetrine	406	526	Violet (405 nm)	525/50

For co-infections in K562-CD209 cells, 250 μl of P1 reporter-virus stock with 2x 2A design (titer in the range of 1–4 x10^5^ FFU/ml) was added to 200,000 cells with a total volume of 850 μl (made up with R10) in 24-well plates. In the case of quadruple co-infections with four different colored viruses, 10 μl of concentrated DENV2-eGFP with titer of 5x10^6^ FFU/ml was used in place of 250 μl of the un-concentrated DENV2-eGFP stock to keep the total volume to 850 μl. The infection was carried out for 45 hours at 37°C, 5% CO_2_, and 80% humidity. The cells were harvested and kept on ice for fluorescent measurement by flow cytometry. For each color of fluorescent DENV2, we used the same flow cytometer settings as in superinfection exclusion analysis.

### Human PBMC infection

500,000 PBMCs were infected with DENV-eGFP with the 2x 2A design at MOI of 0.5 and incubated further at 37°C and 5% CO_2_ for 2 days. Cells incubated without virus were used as negative control. Thereafter, cells were washed and stained with anti-CD3 APC (clone SK7), CD14 PerCP (clone MφP9), and CD19 PE (clone SJ25C1) monoclonal antibodies. The cells were then washed and fixed with 1% formaldehyde in PBS. All antibodies were purchased from BD Pharmingen. Stained cells were analyzed on BD LSR Fortessa. Data analysis was performed using FlowJo software version 10.1. For antibody-dependent enhancement, 1 μg/ml of purified 4G2 (*Flavivirus* E protein specific mAbs) were incubated with virus for one hour at 37 °C before adding to PBMCs (MOI 0.5). Cells incubated with virus but without antibody were used as negative control. After incubation for 2 days, cells were harvested and stained as described in direct DENV infection. Human blood was obtained from donors after providing informed consent, following a protocol (Si439/2007) approved by Siriraj Hospital, Mahidol University, Thailand.

### Infection of reporter DENV2 in C6/36 cells

C6/36 cells were plated at the density of 200,000 cells/well in L15+10% HI-FBS in 24-well plate. One day after seeding, the cells were counted and infected with a DENV2 virus at MOI = 0.1 in 500 μl volume in L15+1.5%HI-FBS. The cells were cultured for 3 days before harvesting for analyses by flow cytometry and immunofluorescence microscopy. For fluorescent microscopy, the cells were fixed with 1X PBS + 3.7% formaldehyde and permeabilized with 1xPBS + 2% Triton-X100. Staining was performed with 4G2 as the primary antibody and Cy3-conjugated anti-mouse IgG (Jackson immunoresearch) as the secondary antibody. The stained cells were imaged under EVOS FL inverted fluorescent microscope (Invitrogen). For flow cytometry, the cells were treated as described above in passaging of reporter DENV2.

### *Aedes aegypti* infection

#### Ethics statement

This study was carried out in strict accordance with the recommendations in the Guide for the Care and Use of Laboratory Animals of the National Institutes of Health. Mice were used only for mosquito rearing as a blood source, according to the protocol (BT-Animal 26/2560). Mosquito infection assays were performed followed the approved protocol (BT-Animal 27/2560). Both animal protocols were approved the BIOTEC Committee for use and care of laboratory animals.

#### Mosquito rearing and maintenance

A colony of *Aedes aegypti* was obtained from the National Institute of Health, Department of Medical Sciences, Thailand. The colony was maintained in BIOTEC’s insectary at 27°C with 80% humidity and a 12-hour day/night, 30-minute dusk/ dawn lighting cycle. The larvae were fed a diet of powdered fish food (Tetrabit). Adults were fed on a 10% sucrose solution supplemented with 2.5% multivitamin syrup (Sevenseas) *ad libitum*.

#### DENV2 infection in *Ae*. *aegypti* by intrathoracic injection

To infect DENV2 in *Ae*. *aegypti* mosquito, 138 nl of virus stock was injected into the thorax of cold-anesthetized 4- to 7-day-old female mosquitoes using a nanoliter injector (Nanoject; Drummond Scientific). The number of injected virus corresponds to approximately 40 FFU/mosquito for DENV2 Clover2-2x 2A, and approximately 800 FFU/mosquito for DENV2 eGFP-2x 2A and the wild-type DENV2-16681. Injected mosquitoes were then maintained on 10% sucrose solution at a condition as mentioned above. Whole mosquito bodies were individually collected at different time points in MEM medium then stored at -80°C before virus titration.

#### Fluorescent imaging of infected mosquito by stereomicroscope

Virus-injected *Ae*. *aegypti* mosquitoes were cold-anesthetized and knocked down with 70% ethanol. The samples were rinsed twice with sterile water. To visualize the fluorescent signal, mosquitoes were squashed with blunt end forceps then imaged under Olympus SZX2-TR30PT fluorescent stereomicroscope with Olympus DP73 digital camera using Olympus Cellsens standard 1.12 software. The mosquitoes were imaged seven days after virus injection.

#### Virus titration of infected mosquitoes by fluorescent and immunostaining focus assay

To determine reporter virus replication in mosquito, virus titers of individual mosquito were determined by fluorescent focus assay on Vero cells. Individual whole mosquito samples were homogenized using acid-washed glass beads, serially diluted, then inoculated onto cells seeded to 80% confluence in 96-well plates. Plates were rocked for 15 min at room temperature, and then incubated for 45 min at 37°C and 5% CO_2_. Subsequently, MEM containing 2% FBS and 1% methylcellulose was added to each well, and plates were incubated for 3 days at 37°C, 5% CO_2_, 80% humidity. Plates were fixed with 4% formaldehyde solution in PBS for 1 hour at 4°C and then washed three times with PBS. Fluorescent focus units were visualized under Olympus IX71 fluorescent inverted microscope. After fluorescent imaging, the plates were stained with 4G2 antibody and goat anti-mouse HRP secondary antibody and visualized with DAB substrate to measure total infectious titers.
